# InSexBase: an annotated genomic resource of sex chromosomes and sex-biased genes in insects

**DOI:** 10.1093/database/baab001

**Published:** 2021-01-28

**Authors:** X i Chen, Yang Mei, Mengyao Chen, Dong Jing, Yumin He, Feiling Liu, Kang He, Fei Li

**Affiliations:** Ministry of Agriculture and Rural Affairs Key Laboratory of Molecular Biology of Crop Pathogens and Insects & Key Laboratory of Biology of Crop Pathogens and Insects of Zhejiang Province, Institute of Insect Sciences, Zhejiang University, Yuhangtang Rd 866, Xihu District, Hanzghou, 310058, China; Ministry of Agriculture and Rural Affairs Key Laboratory of Molecular Biology of Crop Pathogens and Insects & Key Laboratory of Biology of Crop Pathogens and Insects of Zhejiang Province, Institute of Insect Sciences, Zhejiang University, Yuhangtang Rd 866, Xihu District, Hanzghou, 310058, China; Ministry of Agriculture and Rural Affairs Key Laboratory of Molecular Biology of Crop Pathogens and Insects & Key Laboratory of Biology of Crop Pathogens and Insects of Zhejiang Province, Institute of Insect Sciences, Zhejiang University, Yuhangtang Rd 866, Xihu District, Hanzghou, 310058, China; Ministry of Agriculture and Rural Affairs Key Laboratory of Molecular Biology of Crop Pathogens and Insects & Key Laboratory of Biology of Crop Pathogens and Insects of Zhejiang Province, Institute of Insect Sciences, Zhejiang University, Yuhangtang Rd 866, Xihu District, Hanzghou, 310058, China; Ministry of Agriculture and Rural Affairs Key Laboratory of Molecular Biology of Crop Pathogens and Insects & Key Laboratory of Biology of Crop Pathogens and Insects of Zhejiang Province, Institute of Insect Sciences, Zhejiang University, Yuhangtang Rd 866, Xihu District, Hanzghou, 310058, China; Ministry of Agriculture and Rural Affairs Key Laboratory of Molecular Biology of Crop Pathogens and Insects & Key Laboratory of Biology of Crop Pathogens and Insects of Zhejiang Province, Institute of Insect Sciences, Zhejiang University, Yuhangtang Rd 866, Xihu District, Hanzghou, 310058, China; Ministry of Agriculture and Rural Affairs Key Laboratory of Molecular Biology of Crop Pathogens and Insects & Key Laboratory of Biology of Crop Pathogens and Insects of Zhejiang Province, Institute of Insect Sciences, Zhejiang University, Yuhangtang Rd 866, Xihu District, Hanzghou, 310058, China; Ministry of Agriculture and Rural Affairs Key Laboratory of Molecular Biology of Crop Pathogens and Insects & Key Laboratory of Biology of Crop Pathogens and Insects of Zhejiang Province, Institute of Insect Sciences, Zhejiang University, Yuhangtang Rd 866, Xihu District, Hanzghou, 310058, China

## Abstract

Sex determination and the regulation of sexual dimorphism are among the most fascinating topics in modern biology. As the most species-rich group of sexually reproducing organisms on Earth, insects have multiple sex determination systems. Though sex chromosomes and sex-biased genes are well-studied in dozens of insects, their gene sequences are scattered in various databases. Moreover, a shortage of annotation hinders the deep mining of these data. Here, we collected the chromosome-level sex chromosome data of 49 insect species, including 34 X chromosomes, 15 Z chromosomes, 5 W chromosomes and 2 Y chromosomes. We also obtained Y-linked contigs of four insects species—*Anopheles gambiae, Drosophila innubila, Drosophila yakuba* and *Tribolium castaneum*. The unannotated chromosome-level sex chromosomes were annotated using a standard pipeline, yielding a total of 123 030 protein-coding genes, 2 159 427 repeat sequences, 894 miRNAs, 1574 rRNAs, 5105 tRNAs, 395 snoRNAs (small nucleolar RNA), 54 snRNAs (small nuclear RNA) and 5959 other ncRNAs (non-coding RNA). In addition, 36 781 sex-biased genes were identified by analyzing 62 RNA-seq (RNA sequencing) datasets. Together with 5707 sex-biased genes from the *Drosophila* genus collected from the Sex-Associated Gene Database, we obtained a total of 42 488 sex-biased genes from 13 insect species. All these data were deposited into InSexBase, a new user-friendly database of insect sex chromosomes and sex-biased genes.

**Database URL:** http://www.insect-genome.com/Sexdb/.

## Introduction

As the most species-rich metazoans on Earth, insects contain the most variable mechanisms of sex determination in animals, including male heterogamety and female heterogamety (1, 2). Thus, insects form a good model system on which to study the biology of sex, the mechanisms of sex determination and how they evolve (3). In the past decades, investigations of fruit flies, mosquitos and silkworms have yielded copious knowledge in the field of sex determination (4–6). Comparative genomic analyses between insect species have increased our understanding of the evolution of sex chromosomes (7–10). Moreover, understanding insect sex biology has contributed to improvements in both pest control and resource-insect production. For example, by destroying spermatic X chromosomes (11), the sterile insect technique can be used to manipulate sex ratios in vector mosquitoes, thereby dramatically reducing or even crashing their populations (11). In silkworms, a sequence containing an egg color gene is translocated to the W chromosome by radiation treatment. This enables males and females to be easily distinguished before hatching, hence increasing the efficiency of the silk production industry (12). Furthermore, insects display a wide array of sexual dimorphism, including body size, ornamentation and coloration. So far, these have been found to be controlled by different sex-biased genes (13). Understanding how these phenotypic differences arose from sex-biased genes can reveal novel insights into species evolution (14).

Thanks to the rapid development of sequencing technologies, an increasing number of insect sex chromosomes and sex-biased genes have been reported (15–21). Sex-biased genes can be obtained by comparing RNA-seq data from females and males. Studies on sex-biased genes and their functions and uneven chromosomal distribution have drawn more attention recently (22–32). Unfortunately, these data are scattered throughout various genome databases, which often lack annotation information. The Sex-Associated Gene Database (SAGD) catalogs the sex-biased genes of many species, but only a single insect genus, *Drosophila*, is available therein (33). This provides very limited support for in-depth studies of sex biology and sex determination in insects.

To make it easier for researchers to obtain and analyze sex-related genes, we constructed the Insect Sex Chromosome and Sex-Biased Genes Database (InSexBase) by integrating data from 49 sequenced chromosome-level genomes and 62 RNA-seq datasets. InSexBase is intended to provide a comprehensive platform of chromosome-level sex-related genome and gene resources for insects. To the best of our knowledge, InSexBase collects nearly all available chromosome-level insect sex chromosome sequences and sex-biased genes. It offers some widely used web services such as BLAST and JBrowse. Users can browse data by species, sex chromosomes, sex chromosome genes or sex-biased genes. Data grouped by species or chromosome can be downloaded easily, furnishing a valuable resource for researchers in the fields of sex evolution, pest control and public health.

## Materials and methods

### Data collection

**Figure 1.**
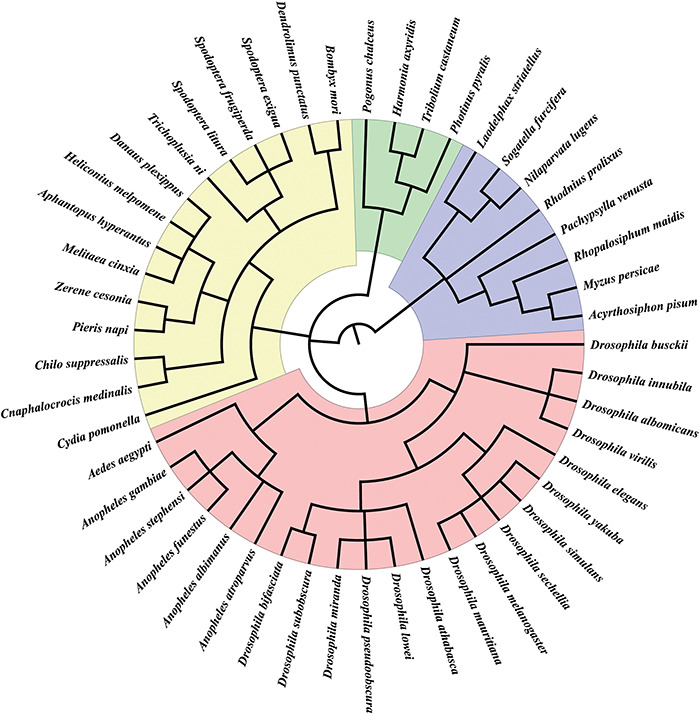
Insect species in InSexBase. InSexBase contains 49 insect species from 28 genera belonging to four orders.

We collected sex chromosome sequences from 49 insect species, including 34 X chromosomes, 15 Z chromosomes, 5 W chromosomes and 2 Y chromosomes from various public databases (Supplemental Table S1). We also obtained Y-linked contigs of four insect species—*Anopheles gambiae, Drosophila innubila, Drosophila yakuba* and *Tribolium castaneum*. Further annotations of Y chromosomes did not include these contigs because they are very short (Supplemental Table S1). These species represent 28 genera in four orders (Figure 1). We also collected 62 RNA-seq datasets from NCBI and EMBL-EBL (Supplemental Table S2) and 5707 sex-biased genes from the *Drosophila* genus from SAGD (http://bioinfo.life.hust.edu.cn/SAGD#!/).

### Gene annotation of sex chromosomes

We obtained the gene annotation information of the genomes with available annotations (Supplemental Table S1). For those genomes without gene annotations, we carried out gene annotation with the following methods.

Repeat sequences were identified by both homology-based and *de novo* prediction methods. For *de novo* predictions, RepeatModeler v1.0.11 was used with default parameters to construct a *de novo* repeat library. For homology-based predictions, RepeatMasker v4.0.7 (34) was used with the Repbase library (35). We used GeneMark-ES (version 4.32) to annotate expected protein-coding genes identified via *de novo* prediction (36). Sequences were BLASTed against the Swiss-Prot database (37) with an *e*-value of 1 × 10^−5^. Then, the Retrieve/ID mapping function (https://www.uniprot.org/uploadlists/) was used to obtain the OrthoDB ID and PDB ID. Gene Ontology analysis was carried out using Blast2GO v5.2 (38). We further mapped these genes to data from the Kyoto Encyclopedia of Genes and Genomes (KEGG) database using the BlastKOALA v2.2 (39) online service (KOALA: KEGG Orthology and Links Annotation). Noncoding RNA genes were predicted by the INFERNAL software against Rfam database, release 14.2 (40). All algorithms were run with default parameters.

### Identifying sex-biased genes

RNA-seq reads were first filtered using Trimmomatic v0.38 (41). The clean reads were then mapped to the corresponding assembled genome using RSEM v1.3.0 (42) to calculate Fragments Per transcripted Kilobase per Million mapped reads (FPKM) and a read count table. Only genes with both male and female FPKM >1 were considered for further analysis. The read count tables for male and female samples were used as input for DESeq2 v1.28.1 (43). Genes were accepted as differentially expressed when DESeq2 returned a *q*-value <0.05 and |Log_2_(Fold Change)| >1. Genes satisfying these conditions comprised the final set of sex-biased genes.

### Database implementation

**Figure 2.**
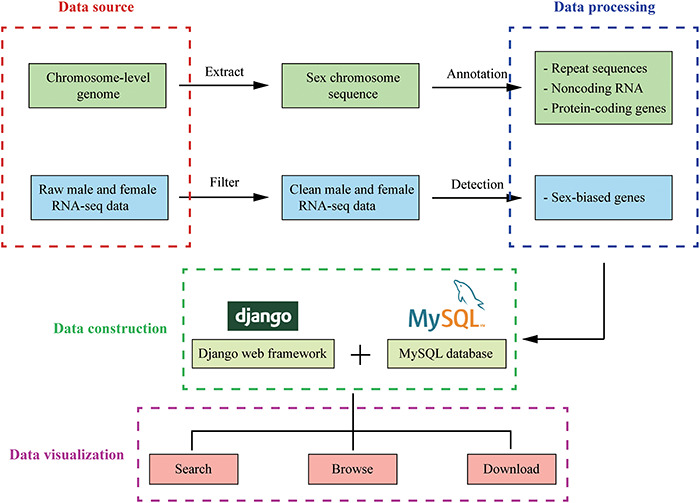
The overview of InSexBase. Genomes and sex-related RNA-seq data were collected from public databases. Sex chromosome sequences were extracted from those genome data. Repeat sequences, non-coding RNAs and protein-coding genes were annotated from sex chromosome. Sex-biased genes were detected from male and female RNA-seq clean data. Finally, these data were deposited in InSexBase built with Django 3.0.5 web framework and all data were stored in a MySQL 5.7.17 database. Users can use browse, search and download functions to explore the database.

The database was built with Django 3.0.5 web framework (https://www.djangoproject.com/), and all data were stored in a MySQL 5.7.17 database (https://dev.mysql.com/) on a CentOS 7.4.1708 web server. Web page templates used Bootstrap framework (https://getbootstrap.com/), Data-Tables (http://datatables.net), jQuery (http://jquery.com) and Bootstrap Table (https://bootstrap-table.com/) libraries to establish a user-friendly front-end interface. On the tools page, the JBrowse Genome Browser (http://www.jbrowse.org/) was used to build a fast and scalable genome browser (Figure 2).

## Results and discussion

### Sex chromosomes in insects

In total, we collected the sex chromosome sequences of 49 insect species from four orders, including 4 Coleoptera species, 8 Hemiptera species, 15 Lepidoptera species and 22 Diptera species (Figure 1).

Lepidopterans are female heterogametic and have both ZW and Z0 systems. InSexBase contains 15 Z chromosomes and 5 W chromosomes of 15 species. Because the W chromosome is packed with repeats (44–48), it remains a difficult problem to obtain a complete W chromosome. The W chromosome sequences of these five species are useful references.

The majority of dipterans have XY sex chromosomes (49). The sex chromosomes differ between various families, however, and there are numerous transitions of sex chromosomes in Diptera (50, 51). Unlike the malaria mosquito *A. gambiae* and *Drosophila* species, *Aedes aegypti* does not have heteromorphic (XY) sex chromosomes (52). The male phenotype determination is by a non-recombining M locus on one copy of autosome 1 (53). InSexBase contains 22 X chromosomes of 22 species in Diptera and 1 Y chromosome of *D. melanogaster*. InSexBase also contains Y-linked contigs of three Diptera species, *A. gambiae, D. innubila* and *D. yakuba. Aedes aegypti* has a homomorphic sex chromosome system, and chromosome 1 is regarded as the X chromosome in InSexBase.

The majority of sexually reproducing beetles are male heterogametic (54). Unlike dipterans and lepidopterans, the base genetic mechanism of sex determination has not been identified for beetles (49). InSexBase contains four X chromosomes from four species in Coleoptera and Y-linked contigs of *T. castaneum*. Of these, *Photinus pyralis* has an X0 system, and the other three have XY systems (54).

Most hemipteran insects employ the XY or X0 sex determination system, while most aphids reproduce through cyclic parthenogenesis (49). InSexBase contains eight X chromosomes from eight species in Hemiptera (five aphids and three plant hoppers) and one Y chromosome from the plant hopper *Nilaparvata lugens*.

### Repeat sequences in sex chromosomes

**Figure 3.**
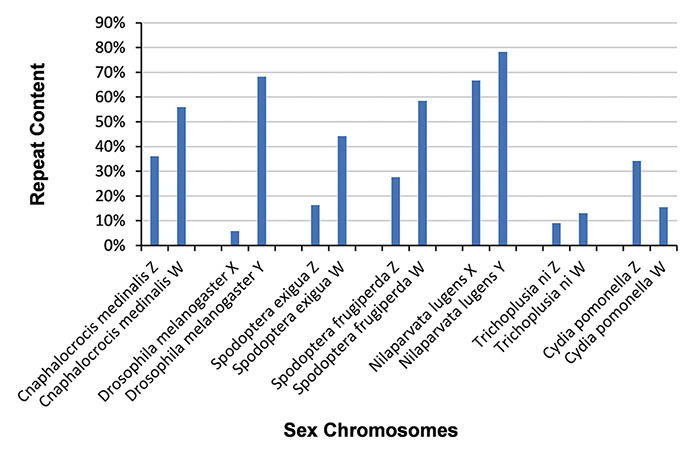
Distribution of repeat sequences in 14 sex chromosomes. Sex chromosomes generally contain a high percentage of repeat sequences.

We identified repeat sequences in 55 sex chromosomes with RepeatModeler v1.0.11 and RepeatMasker v4.0.7 (34), yielding 2 159 427 repeat sequences. The evolution of sex-limited chromosomes is often accompanied by the acquisition of repetitive DNA (44–48). The occurrence of repeat sequences in the W and Y chromosomes of six species (*Cnaphalocrocis medinalis, D. melanogaster, Spodoptera exigua, Spodoptera frugiperda, N. lugens* and *Trichoplusia ni*) is much higher than that in their Z and X chromosomes (Figure 3). However, repeat sequences in the W chromosome of *Cydia pomonella* are not particularly frequent (Figure 3).

### Protein-coding genes and non-coding RNA genes in sex chromosomes

To collect protein-coding genes and non-coding RNA genes in sex chromosomes, we obtained the gene annotation information of the genomes with available annotations. For those genomes without gene annotations, we carried out gene annotation as described in the ‘Materials and methods’ section. Consequently, a total of 123 030 protein-coding genes, 894 miRNAs, 1574 rRNAs, 5105 tRNAs, 395 snoRNAs, 54 snRNAs and 5959 other ncRNAs were predicted on 56 sex chromosomes in 49 species. The 34 X chromosomes were predicted to contain 101 060 protein-coding genes, 593 miRNAs, 1527 rRNAs, 4071 tRNAs, 389 snoRNAs, 54 snRNAs and 5773 other RNAs. The 15 Z chromosomes contain 16 799 protein-coding genes, 286 miRNAs, 26 rRNAs, 757 tRNAs, 6 snoRNAs and 59 other RNAs; 2 Y chromosomes contain 354 protein-coding genes, 11 miRNAs, 12 rRNAs and 236 tRNAs. The five W chromosomes contain 4817 protein-coding genes, 4 miRNAs, 9 rRNAs, 95 tRNAs and 14 other RNAs (more information provided in Supplemental Table S1).

In *D. melanogaster*, four X-related proteins encoded by the *scute, sisA, runt* and *unpaired* genes serve as the primary determinants of X dose (55–59), which plays an important role in sex determination in fruit flies. In the silkworm, a W-linked piRNA (PIWI-interacting RNA) is the primary determiner of sex (4). The protein-coding genes and non-coding RNA genes on the sex chromosomes may also be important for sex determination in non-model insects.

### Sex-biased genes in insects

We analyzed 62 RNA-seq datasets to deduce sex-biased genes with RSEM v1.3.0 (42) and DESeq2 (43), yielding 36 781 sex-biased genes. We also downloaded the 5707 identified sex-biased genes of three *Drosophila* species from SAGD. Finally, we obtained a total of 42 488 sex-biased genes from 13 insect species from seven genera in four orders. The study of sex-biased genes is important not only for understanding gene regulation and evolution but also for their application to insect reproduction and pest control (14, 60–63).

### Web interface and usage

InSexBase provides a user-friendly interface for searching, browsing and downloading data about insect sex chromosome and sex-biased genes. In the top navigation bar, there are eight units including ‘Home’, ‘Species’, ‘Dataset’, ‘Tools’, ‘Download’, ‘Help’, ‘Links’ and ‘About us’. The ‘Home’ page offers an introduction to InSexBase, a quick gene search function and some interface entrances. By clicking the left-side logo of the Webpage, users can find some insect pictures (Supplemental Table S3).

**Figure 4.**
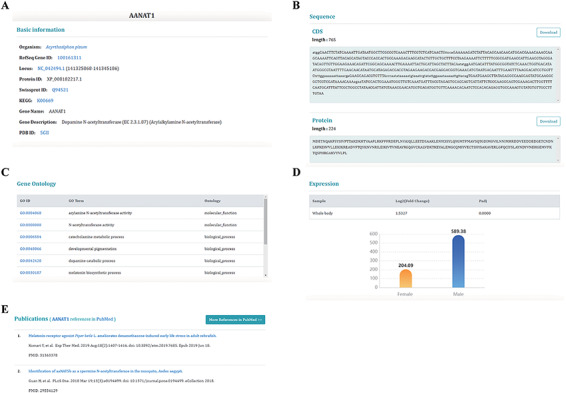
Information on sex-biased genes. (A) Basic genetic information. (B) CDS and protein sequences. (C) The Gene Ontology information of this gene. (D) The expression of sex-biased genes. (E) Publications relating to this gene.

There is an unrooted phylogenetic tree in the ‘Species’ unit for users to browse species information and download data. The ‘Dataset’ unit currently includes five pages for users to browse data about sex chromosomes, sex chromosomal genes, ncRNAs, repeat information and sex-biased genes. The page dedicated to genes shows the most detailed information about genes (Figure 4). The database provides two bioinformatics tools: BLAST and JBrowse. Users can use ‘BLAST’ to search target genes and use ‘JBrowse’ to browse genome information. We provide convenient downloading options in the ‘Download’ unit to download anything from the database. The ‘Help’ unit offers detailed instructions and the ‘Links’ unit provides links to related databases.

### Future directions

1. Synteny analysis is helpful in studying the evolution of sex chromosomes. We plan to add online synteny analysis to InSexBase.
2. Due to the rapid development of sequencing technology in recent years, the genomes of an increasing number of insects will be sequenced and their sex-related gene data will be identified. We plan to update InSexBase periodically to keep the database up-to-date.
3. We will add more expression evidence by integrating RNA-seq data into InSexBase.
4. 3D genomics, which explores the crucial role the three-dimensional configuration of the genome has in gene regulation, will be added in the future to study the genomic structure of sex chromosomes.

## Conclusion

To the best of our knowledge, InSexBase is the first database of sex-related chromosomes, genomes and gene resources in insects. By unifying and expanding insect sex information, InSexBase will be extremely useful for evolution analysis and comparative genomics in sex evolution, pest control, mosquito biology, resource-insect management and public health.

## Supplementary Material

baab001_Supp
